# Regulation of biomass growth and carbon partitioning in poplar – molecular characterization of a candidate gene

**DOI:** 10.1186/1753-6561-5-S7-P122

**Published:** 2011-09-13

**Authors:** Cintia Ribeiro, Evandro Novaes, Christopher Dervinis, Matias Kirst

**Affiliations:** 1 Plant Molecular and Cellular Biology Graduate Program, University of Florida, Gainesville, FL, 32611, USA; 2 Escola de Agronomia e Eng. de Alimentos, Federal University of Goias, Goias, Brazil; 3 School of Forest Resources and Conservation, Institute of Food and Agricultural Sciences, University of Florida, Gainesville, FL, 32611, USA

## 

Improvement of plant feedstock for bioenergy production can be achieved by modifying wood chemical properties and increasing biomass productivity. We previously identified a candidate gene for carbon partitioning and growth on chromosome 13 (cpg13) of poplar. Cpg13 was identified as the regulator of carbon partition and growth within a QTL interval that explains 56% of the variation in cellulose to lignin ratio, as well as 20-25% of the heritable variation in biomass. Putative homologues of cpg13 in Arabidopsis are annotated as proteins of unknown function; therefore, the functional characterization of cpg13 is essential. At present, evidence of the functional role of cpg13 is being obtained by the analysis of poplar transgenic plants transformed with RNAi, overexpression and GFP-fused – cpg13 constructs. Preliminary data indicates that down-regulating cpg13 positively impacts growth, while a negative effect is detected in lines overexpressing cpg13. Transgenic poplar 35S::cpg13:GFP shows localization in cell wall, consistent with *in silico* predictions. Comparative genomics indicate moderate similarity with methyltransferase. Analysis of the impact of differentially regulating cpg13 on lignin and cellulose is currently under way in the mutants. Furthermore, attempts to purify the cpg13 protein are in progress, to define its role through a series of biochemical function assays.

